# Angio-Long Noncoding RNA MALAT1 (rs3200401) and MIAT (rs1061540) Gene Variants in Ovarian Cancer

**DOI:** 10.3390/epigenomes8010005

**Published:** 2024-01-29

**Authors:** Manal S. Fawzy, Afaf T. Ibrahiem, Dalia Mohammad Osman, Amany I. Almars, Maali Subhi Alshammari, Layan Tariq Almazyad, Noof Daif Allah Almatrafi, Renad Tariq Almazyad, Eman A. Toraih

**Affiliations:** 1 Department of Biochemistry, Faculty of Medicine, Northern Border University, Arar 73213, Saudi Arabia; 2 Unit of Medical Research and Postgraduate Studies, Faculty of Medicine, Northern Border University, Arar 73213, Saudi Arabia; 3 Department of Pathology, Faculty of Medicine, Northern Border University, Arar 73213, Saudi Arabia; 4 Department of Medical Laboratories Technology, Faculty of Applied Medical Sciences, Northern Border University, Arar 73213, Saudi Arabia; 5 Department of Medical Laboratory Sciences, Faculty of Applied Medical Sciences, King Abdulaziz University, Jeddah 21589, Saudi Arabia; aialmars@kau.edu.sa; 6 Hematology Research Unit, King Fahd Medical Research Center, King Abdulaziz University, Jeddah 21589, Saudi Arabia; 7 Faculty of Medicine, Northern Border University, Arar 73213, Saudi Arabia; maalix1@gmail.com (M.S.A.); laaltamimii7@gmail.com (L.T.A.); almatrafunoof@gmail.com (N.D.A.A.); 8 Faculty of Applied Medical Sciences, Northern Border University, Arar 73213, Saudi Arabia; renadalmazyad57@gmail.com; 9 Division of Endocrine and Oncologic Surgery, Department of Surgery, School of Medicine, Tulane University, New Orleans, LA 70112, USA

**Keywords:** ovarian cancer, angio-lncRNAs, MIAT, MALAT, gene variant, single-nucleotide polymorphism, allelic discrimination, TaqMan real-time PCR

## Abstract

The genotyping of long non-coding RNA (lncRNA)-related single-nucleotide polymorphisms (SNPs) could be associated with cancer risk and/or progression. This study aimed to analyze the angiogenesis-related lncRNAs MALAT1 (rs3200401) and MIAT (rs1061540) variants in patients with ovarian cancer (OC) using “Real-Time allelic discrimination polymerase chain reaction” in 182 formalin-fixed paraffin-embedded (FFPE) samples of benign, borderline, and primary malignant ovarian tissues. Differences in the genotype frequencies between low-grade ovarian epithelial tumors (benign/borderline) and malignant tumors and between high-grade malignant epithelial tumors and malignant epithelial tumors other than high-grade serous carcinomas were compared. Odds ratios (ORs)/95% confidence intervals were calculated as measures of the association strength. Additionally, associations of the genotypes with the available pathological data were analyzed. The heterozygosity of MALAT1 rs3200401 was the most common genotype (47.8%), followed by C/C (36.3%). Comparing the study groups, no significant differences were observed regarding this variant. In contrast, the malignant epithelial tumors had a higher frequency of the MIAT rs1061540 C/C genotype compared to the low-grade epithelial tumor cohorts (56.7% vs. 37.6, *p* = 0.031). The same genotype was significantly higher in high-grade serous carcinoma than its counterparts (69.4% vs. 43.8%, *p* = 0.038). Multivariate Cox regression analysis showed that the age at diagnosis was significantly associated with the risk of OC development. In contrast, the MIAT T/T genotype was associated with a low risk of malignant epithelial tumors under the homozygote comparison model (OR = 0.37 (0.16–0.83), *p* = 0.017). Also, MIAT T allele carriers were less likely to develop high-grade serous carcinoma under heterozygote (CT vs. CC; OR = 0.33 (0.12–0.88), *p* = 0.027) and homozygote (TT vs. CC; OR = 0.26 (0.07–0.90), *p* = 0.034) comparison models. In conclusion, our data provide novel evidence for a potential association between the lncRNA MIAT rs1061540 and the malignant condition of ovarian cancer, suggesting the involvement of such lncRNAs in OC development.

## 1. Introduction

Ovarian cancer (OC) represents one of the most common gynecological malignancies that has a high mortality rate worldwide [1,2]. It is ranked within the top 10 cancers with high age-standardized incidences and mortality rates according to global cancer statistics [3,4]. According to a recent report, the “adjusted years of life with disabilities (DALYs)” due to OC were reported to be 5,359,737, of which 5,205,660 were related to “lost years of life”, and 154,077 were related to “years of life with disabilities” [5].

Based on the histopathological, molecular, and genetic profiles, malignant epithelial ovarian cancer consists of five main types: “high-grade serous (HGSC), endometrioid (EC), clear cell (CCC), mucinous (MC) and low-grade serous (LGSC)” carcinomas [6]. Although the etiopathology of OC is multifactorial, including genetic and epigenetic (the phenotypic alteration in gene expression without the modification of the DNA sequence itself) factors, proto-oncogenes/tumor suppressor genes, dysregulation plays a significant role in tumorigenesis [7]. Extensive molecular research has identified some risk factors for ovarian cancer, including BRCA1, BRCA2, and Lynch syndrome [8]. Nevertheless, the incidence of these genetic mutations is relatively low, and they only explain a small fraction of cases [9]. Therefore, there is a growing interest in identifying new genetic variants that may contribute to ovarian carcinogenesis.

Over the past decade, long non-coding RNAs (lncRNAs; >200 nucleotides in length) have emerged as critical players in gene regulatory networks by influencing the chromatin dynamics, transcriptional control, post-transcriptional processing, and intracellular signaling pathways [10]. Several studies have unveiled that lncRNAs contribute to the oncogenic landscape of OC by regulating the hallmarks of cancer, such as sustained proliferative signaling, resistance to cell death, angiogenesis, and metastasis [11,12]. They act as molecular scaffolds, guides, and decoys that can recruit chromatin-modifying enzymes, thereby altering the epigenetic state of specific gene loci [11,13,14]. With their ability to impact the expressions of genes involved in cell cycle regulation, apoptosis, and the epithelial-to-mesenchymal transition, lncRNAs are implicated in the modulation of the cellular phenotypes seen in cancer, including OC [15,16].

Moreover, the aberrant expression profiles of lncRNAs in ovarian tumors compared to normal tissues present them as potential biomarkers for early detection and prognosis [17,18]. Studies have also highlighted the functional diversity of lncRNAs, with some exhibiting tumor-suppressive activities and others acting as oncogenic promoters [19,20]. Furthermore, the interaction of lncRNAs with miRNA molecules adds a layer of post-transcriptional regulation, where lncRNAs can serve as competing endogenous RNAs (ceRNAs) or “miRNA sponges” to modulate the miRNA activity on target mRNA transcripts [21]. This complex interaction network significantly impacts the cellular milieu and contributes to the phenotypic outcomes associated with cancer progression and therapeutic resistance [15,22,23].

Abnormal angiogenesis is associated with the etiopathology of several disorders, including cancer [24]. Accumulating evidence has revealed essential roles for ncRNAs in angiogenesis and identified a group of lncRNAs termed “Angio-lncRs”, which regulate or are associated with angiogenesis [25]. For example, “metastasis-associated lung adenocarcinoma transcript 1 (MALAT1)” is vital for angiogenesis through its regulation of the cyclins and cell cycle inhibitors, as evidenced by the reduced retinal vascularization in MALAT1^−/−^ mice [26]. Also, the lncRNA “myocardial infarction-associated transcript (MIAT)” functions as a sponge for microRNA-150-5p, relieving the miR-150-5p repression of the vascular endothelial growth factor (VEGF), and its knockdown has been reported to decrease endothelial cell proliferation and migration and vascular network formation in vitro [27]. 

Interestingly, in their review article, Minotti et al. stated that MALAT1and MIAT presented a higher number than expected of function-impacting somatic mutations, and they confirmed that the single-nucleotide polymorphisms (SNPs) of lncRNAs might play a role in carcinogenesis, supporting their use as genetic biomarkers [28]. Several studies have highlighted the association between the genetic variants and SNPs that occur in regions transcribed into the lncRNAs and increase the risk and/or progression of cancers [29,30,31,32,33,34,35,36]. These SNPs may influence lncRNA regulation and the process of splicing and/or stability, resulting in the modification of its interacting partners, and thus they could be correlated with tumorigenesis [37,38,39]. Based on (1) the screening of dbSNPs (www.ncbi.nlm.nih.gov) (last accessed 20 January 2023) for a “minor allele frequency (MAF)” ≥ 0.1, (2) a literature review of the functional evidence of the selected variants, and/or (3) the fact that no previous studies have investigated the association of the selected SNPs with OC progression, two angio-lncRNA-related candidate SNPs were selected (i.e., MALAT rs3200401 and MIAT rs1061540) to explore their association with OC progression and/or the OC phenotype and correlate the results to the available clinicopathological variables. The results of this study can help with the risk stratification of patients with benign and/or borderline tumors and could help to improve the prognostic strategies for patients with OC and, subsequently, their individualized therapeutic management in the near future.

## 2. Results

### 2.1. Characteristics of the Study Population

**Figure 1:**
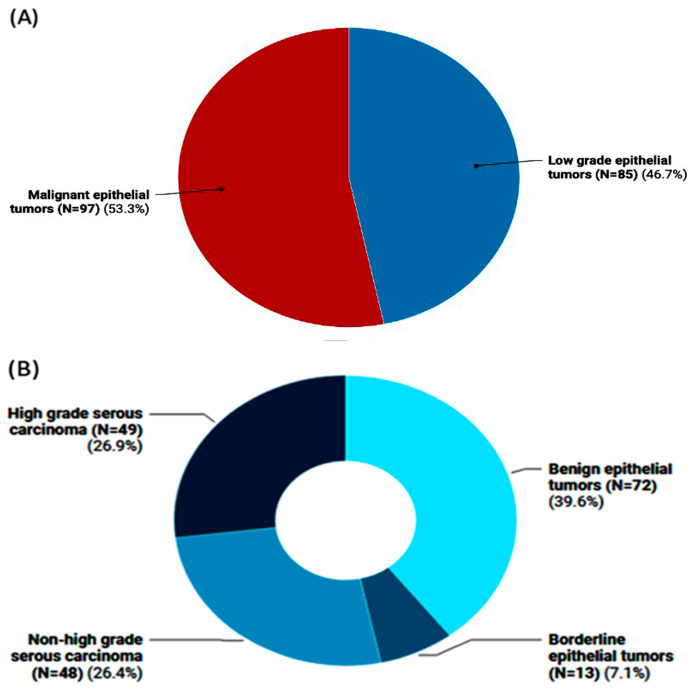
Frequency of ovarian tumors in the study population. (**A**) Classification of ovarian tumors into malignant epithelial tumors vs. low-grade ones (i.e., benign and borderline cases). (**B**) Subclassification of ovarian tumors into high-/non-high-grade serous carcinomas that pertain to malignant OC and the non-malignant types (i.e., benign and borderline epithelial tumor cases).

The study included 182 female patients with a mean age of 44.4 ± 15.01 years. Of them, 97 patients (53.3%) were diagnosed with malignant epithelial tumors with an average age of 47.1 ± 13.8 years, compared to 85 women (46.7%) who had low-grade epithelial tumors with an average age of 41.3 ± 15.7 years (Figure 1A). Among the low-grade epithelial tumors, 13 instances had borderline epithelial tumors, and 72 cases were benign epithelial tumors. High-grade serous carcinoma was observed in 49 individuals with malignant epithelial tumors, while non-high-grade serous carcinoma was observed in 48 cases (Figure 1B).

Table 1 presents the demographic and pathological characteristics of the study population, encompassing a total of 182 tumors, with 85 low-grade epithelial tumors and 97 malignant epithelial tumors. The demographic data show that age is significantly associated with the tumor types (*p* = 0.001). Among patients under 30 years, 18.7% had tumors, with 30.6% in the low-grade epithelial group and 8.2% in the malignant epithelial group. Among patients aged 30–49 years, 52.7% had tumors, with 44.7% in the low-grade epithelial group and 59.8% in the malignant epithelial group. Lastly, among patients aged 50 years and above, 28.6% had tumors, with 32.0% in the low-grade epithelial group and 24.7% in the malignant epithelial group.

### 2.2. Pathological and Molecular Assessment

The pathological subtypes showed significant differences between the low-grade and malignant epithelial tumor groups (*p* < 0.001). The IHC staining results demonstrated significant differences between the tumor groups for the human epidermal growth factor receptor 2 (HER2) protein staining (*p* < 0.001) and P53 protein staining (*p* < 0.001). For HER2, 53.3% were negative, 13.2% were 1+, 14.8% were 2+, and 18.7% were 3+. For P53, 73.6% were negative, and 26.4% were positive. The KRAS protein staining also showed a significant difference (*p* = 0.003), with 53.3% negative and 46.7% positive. However, no significant difference was observed for the EGFR protein staining (*p* = 0.45), with 96.2% (175 cases) negative and 3.8% (7 cases) positive (Table 1).

The gene mutation screening results did not show significant differences for *BRAF* V600 (*p* = 0.24) or *KRAS* codon 12 (*p* = 0.54). For *BRAF* V600, 8.8% were wild, 84.6% were heterozygote, and 6.6% were mutant. For *KRAS* exon 12, 15.4% were wild, and 84.6% were mutant. *KRAS* exon 13 was wild for all patients in the study (Table 1).

### 2.3. Subgroup Analysis of Malignant Epithelial Tumors

Table 2 highlights the molecular and demographic differences between high-grade serous carcinoma and other malignant epithelial tumors. There is no statistically significant difference in the age distribution of patients with high-grade serous carcinoma versus those with other malignant epithelial tumors (*p* = 0.27).

The IHC staining results display no significant differences between high-grade serous carcinoma and other malignant epithelial tumors for the HER2 protein staining (*p* = 0.28) and EGFR protein staining (*p* = 0.36). However, a remarkable disparity is evident in the P53 protein staining (*p* < 0.001), with 100% (49 cases) of high-grade serous carcinoma patients being P53-positive and 100% (48 cases) of patients with other malignant epithelial tumors being P53-negative.

No significant differences were found in the distribution of the KRAS protein staining between high-grade serous carcinoma and other malignant epithelial tumors (*p* = 0.67).

The gene mutation analysis reveals no significant differences in the distribution of the *BRAF* V600 mutation status (*p* = 0.15) between the two tumor types. Nevertheless, a significant difference is observed for the *KRAS* exon 12 mutation status (*p* = 0.007), with 77.1% (37 cases) of high-grade serous carcinoma patients having a mutant allele, compared to 95.9% (47 cases) among those with other malignant epithelial tumors.

### 2.4. In Silico Data Analysis and Variant Functional Annotation

**Figure 2:**
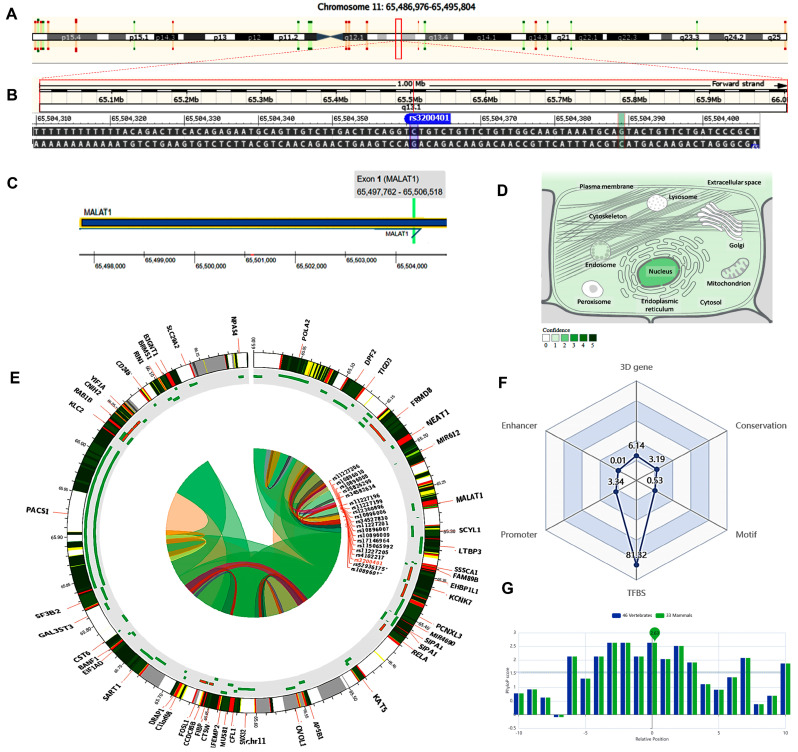
Structural analysis of the MALAT1 gene and related 3D interactions with other genes/variants mediated via chromatin loops. (**A**) MALAT1 is located on the long arm of chromosome 11: 65,497,688−65,506,516 on the forward strand according to the GRCh38.p14 assembly. (**B**) The studied non-coding transcript variant rs3200401 (C/T) is located on position 11:65504361 (highlighted) with the “C” ancestral nucleotide substituted with the alternative (minor allele) “T” (http://asia.ensembl.org/, accessed on 20 March 2023) (https://www.ncbi.nlm.nih.gov/snp/rs3200401, accessed on 20 March 2023). (**C**) The rs3200401 (C/T) is in exon one of the MALAT transcript (https://www.thermofisher.com/, accessed on 20 March 2023). (**D**) MALAT1 subcellular distribution. The degree of color is correlated to its cellular abundance (https://www.genecards.org/, accessed on 20 March 2023). (**E**) A Circos plot showing the chromatin loops and other 2D signatures of the variant of interest as depicted using 3DSNP 2.0 (https://omic.tech/3dsnpv2/, accessed on 20 March 2023). In the plot, from the outside to the inner side, the circle represents “the chromatin states, annotated genes, the current SNP of interest and associated SNPs, and 3D chromatin interactions”, respectively. The color key of the chromatin states (n =15) and chromatin loops in twelve cell types are shown in Figures S1 and S2, respectively. (**F**) The radar chart shows the distribution of the six scores related to the variant of interest. (**G**) The conservation score of the variant of interest is 2.63, calculated from vertebrate (n = 46) and mammal (n = 33) genome multiple alignments.

The MALAT1 gene (ENSG00000251562) is located on chromosome 11q13.1: 65,497,688−65,506,516 (forward strand, Figure 2A) and has 17 transcripts. It is implicated in the positive regulation of cell motility (gene ontology (GO): 2000147). The studied variant rs3200401 (NC_000011.10:g.65504361C>T, Figure 2B) is present in the first exon of MALAT1 (Figure 2C), overlapping seven MALAT1 transcripts. The predominant subcellular localization of MALAT1 with a high confidence level is in the nucleus (Figure 2D). A customizable Circos plotting system was developed using 3DSNP 2.0 (https://omic.tech/3dsnpv2/) (accessed 30 March 2023) to display the 3D chromatin topology and a set of important chromatin marks surrounding the SNP of interest. The Circos tracks from outside to inside represent the chromatin states, RefSeq genes, DNase I hypersensitive sites (DHSs) and histone modifications, transcription factor binding sites (TFBSs), associated SNPs, and chromatin loops (Figure 2E). The ovarian tissue was selected for the cell type option in the case of histone modification yield, and the epithelial cells were selected for the transcriptional factor prediction. The related comprehensive scoring system to assess the functionality of rs3200401 in different aspects is depicted in Figure 2F. This SNP interacts with 16 genes via chromatin loops with a corresponding score of 6.14. It is located in the Enhancer state in one cell type, with a score of 0.01, and in the Promoter state in eight cell types, with a score of 3.34. Also, it is located in 71 transcription factor binding sites (TFBSs), with a score of 81.32. Its PhyloP conservation score is 2.63, with a corresponding score of 3.19. The total functionality score of this SNP is 94.53 (Figure 2F). The “PhyloP score” absolute values represent “-log(*p*-value) under a null hypothesis of neutral evolution”. Positive scores are assigned for predicted conserved sites, while negative scores are assigned for sites predicted to be fast-evolving.

**Figure 3:**
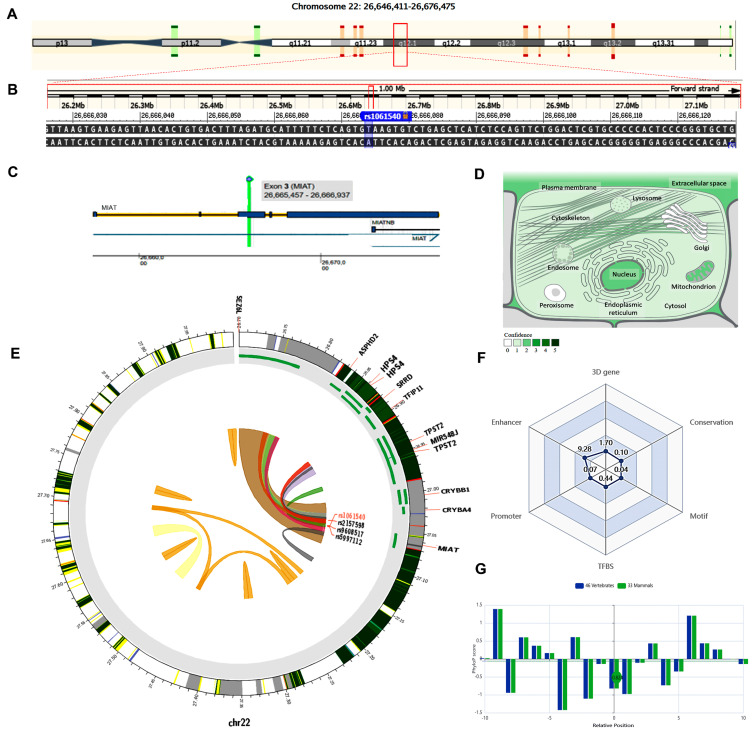
Structural analysis of MIAT gene and related 3D interactions with other genes/variants mediated via chromatin loops. (**A**) MIAT is located on the long arm of chromosome 22: 26,646,411−26,676,475 on the forward strand, according to the GRCh38.p14 assembly. (**B**) The studied non-coding transcript variant rs1061540 (T/C) is located on position 22: 27062037 (highlighted) with the “T” ancestral nucleotide substituted with the alternative (minor allele) “C” (http://asia.ensembl.org/, accessed on 20 March 2023) (https://www.ncbi.nlm.nih.gov/snp/rs1061540, accessed on 20 March 2023). (**C**) The rs1061540 (T/C) is in the third exon of the MIAT transcript (https://www.thermofisher.com/, accessed on 20 March 2023). (**D**) MIAT subcellular distribution. The degree of color is correlated to its cellular abundance (https://www.genecards.org/, accessed on 20 March 2023). (**E**) A Circos plot showing the chromatin loops and other 2D signatures of the variant of interest as depicted using 3DSNP 2.0 (https://omic.tech/3dsnpv2/, accessed on 20 March 2023). In the plot, from the outside to the inner side, the circle represents “the chromatin states, annotated genes, the current SNP of interest and associated SNPs, and 3D chromatin interactions”, respectively. The color key of the chromatin states (n = 15) and chromatin loops in twelve cell types are shown in Figures S1 and S2, respectively. (**F**) The radar chart shows the distribution of the six scores related to the variant of interest. (**G**) The conservation score of the variant of interest is −0.824, calculated from vertebrate (n = 46) and mammal (n = 33) genome multiple alignments.

The MIAT gene (ENSG00000225783) is on chromosome 22q12.1: 26,646,411−26,676,475 (forward strand, Figure 3A) and has 30 transcripts. It is implicated in cell fate specification (gene ontology (GO): 0001708). The studied variant rs1061540 (NC_000022.11:g.26666074T>C, Figure 3B) is present in the third exon of MIAT (Figure 3C), overlapping 12 MIAT transcripts. The predominant subcellular localization of MIAT with a high confidence level is in the nucleus (Figure 3D). A customizable Circos plotting system was developed using 3DSNP 2.0 (https://omic.tech/3dsnpv2/) (accessed 30 March 2023) to display the 3D chromatin topology and a set of essential chromatin marks surrounding the SNP of interest (Figure 3E). The ovarian tissue was selected for the cell type option in case of histone modification yield, and the epithelial cells were selected for the transcriptional factor prediction. The related comprehensive scoring system to assess the functionality of rs1061540 in different aspects is depicted in Figure 3F. This SNP interacts with nine genes via chromatin loops with a corresponding score of 1.70. It is located in the Enhancer state in 31 cell types, with a score of 9.28, and in the Promoter state in one cell type, with a score of 0.07. Also, it locates in three TFBSs with a score of 0.44. Its PhyloP conservation score is −0.824, with a corresponding score of 0.10. The total functionality score of this SNP is 11.63 (Figure 3F). As the absolute values of the related PhyloP scores are negative, it is predicted to be a fast-evolving variant, as explained previously.

According to the tissue-specific “RegVar model”, the extents to which the studied two SNPs are likely to affect the regulation of the corresponding gene in the selected tissue (i.e., ovary) are 0.219 and 0.641 for the MALAT1 rs3200401 and MIAT rs1061540 variants, respectively (Table S1).

### 2.5. MALAT1 (rs3200401) Variant Genotype and Allele Frequencies in Patients with Ovarian Tumors

**Figure 4:**
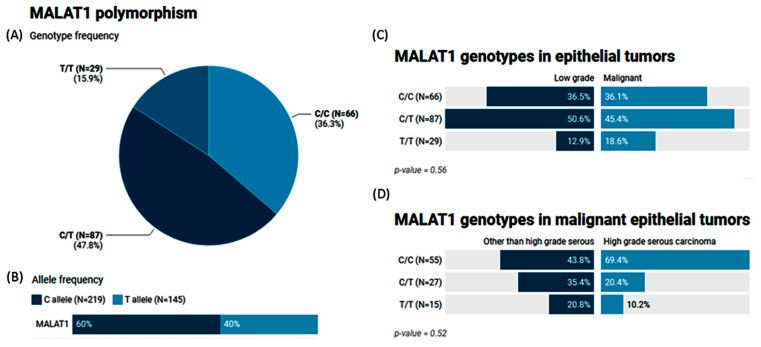
Genetic analysis of MALAT1 rs3200401 T/C genetic variant. Values are shown as numbers and/or percentages. (**A**) Genotype frequency in the overall study population. (**B**) Allele frequency in the overall study population. (**C**) Genotype frequency stratified by types of ovarian tumors (benign/borderline vs. malignant). (**D**) Genotype frequency stratified by types of malignant OC (high-grade serous vs. others). The chi-square test was applied. Statistical significance was set at *p* < 0.05.

The MAF (T allele) of MALAT1 rs3200401 accounted for 0.40 of the study population. Heterozygosity was the most common genotype (47.8%), followed by C/C (36.3%) (Figure 4A,B). In a comparison between the study groups, no significant differences were observed (*p* = 0.56 and 0.52) (Figure 4C,D).

### 2.6. The Impact of the MALAT1 (rs3200401) Variant on Ovarian Cancer Risk

As depicted in Table 3, there is no significant impact of the MALAT1 (rs3200401) gene variant on the OC risk using AIC and BIC measurements.

### 2.7. Genotype and Allele Frequencies of the MIAT (rs1061540) Variant in Patients with Ovarian Tumors

**Figure 5:**
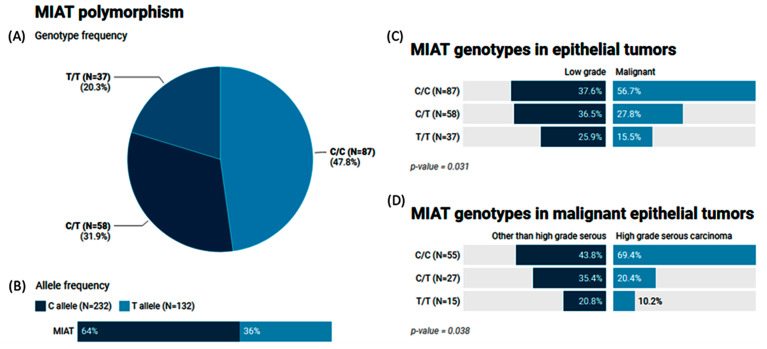
Genetic analysis of MIAT rs1061540 T/C genetic variant. Values are shown as numbers and/or percentages. (**A**) Genotype frequency of MIAT gene in the study population. (**B**) Allele frequency of MIAT single-nucleotide polymorphism. (**C**) Comparison of genotype frequencies between malignant and low-grade epithelial tumors. (**D**) Genotype frequencies in malignant epithelial tumors according to histological variant. The chi-square test was applied. Statistical significance was set at *p* < 0.05.

The MAF (T allele) of MIAT rs1061540 accounted for 0.36 of the study population. The genotype frequencies for C/C, C/T, and T/T were 47.8%, 31.9%, and 20.3%, respectively (Figure 5A,B). Our results showed that malignant epithelial tumors had a higher frequency of the C/C genotype compared to the low-grade epithelial tumor cohorts (56.7% vs. 37.6, *p* = 0.031). The same genotype was significantly higher in high-grade serous carcinoma than its counterparts (69.4% vs. 43.8%, *p* = 0.038) (Figure 5C,D).

### 2.8. The Impact of the MIAT (rs1061540) Variant on Ovarian Cancer Risk

As depicted in Table 4, MIAT rs1061540 shows an association with the OC risk under the homozygous genetic model, as the TT genotype was associated with a lower OC risk (OR (59% CI): 0.39 (0.18–0.88), *p* = 0.040), and under the dominant model (0.47 (0.26–0.87), *p* = 0.014).

### 2.9. Genotype Combination and Ovarian Cancer Risk

None of the examined genotype combinations (TC, CT, and TT) exhibit a statistically significant association with the OC risk when compared to the reference genotype combination (CC) (Table 5).

### 2.10. Multivariate Cox Regression Analysis

**Figure 6:**
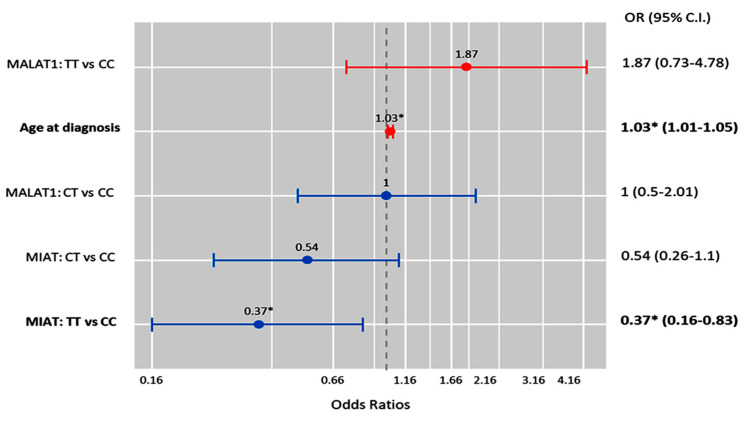
Factors predicting malignant ovarian cancer. Cox regression analysis was used. Results were reported as odds ratios (ORs) and 95% confidence intervals (CIs). The red bar indicates an OR > 1.0, while the blue bar indicates an OR ≤ 1.0. * Statistical significance was set at *p* < 0.05 (bold values).

Multivariate Cox regression analysis showed that the age at diagnosis was significantly associated with the risk of OC development (OR = 1.03 (1.0–1.05), *p* = 0.011), while the MIAT T/T genotype was associated with a low risk of malignant epithelial tumors under the homozygote comparison model (OR = 0.37 (0.16–0.83), *p* = 0.017) (Figure 6).

**Figure 7:**
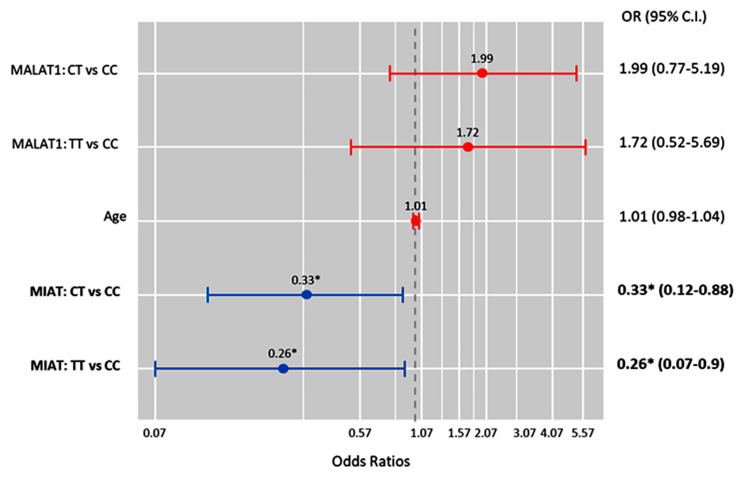
Factors predicting high-grade serous carcinoma. Cox regression analysis was performed. Results were reported as odds ratios (ORs) and 95% confidence intervals (CIs). The red bar indicates an OR > 1.0, while the blue bar indicates an OR < 1.0. * Statistical significance was set at *p* < 0.05 (bold values).

Also, MIAT T allele carriers were less likely to develop high-grade serous carcinoma under the heterozygote (CT vs. CC; OR = 0.33 (0.12–0.88), *p* = 0.027) and homozygote (TT vs. CC; OR = 0.26 (0.07–0.90), *p* = 0.034) comparison models (Figure 7).

## 3. Discussion

Ovarian carcinoma, while not leading in incidence compared to cervical and endometrial cancers, is unfortunately recognized as the most lethal malignancy of the female reproductive system. The majority of the advancements in the diagnostic/prognostic factors for this malignancy are currently steered by the identification of novel biomarkers derived from blood or tissue specimens, which could significantly influence management strategies, with an end goal of optimizing patient outcomes and ultimately decreasing the mortality rates attributable to this challenging disease [41].

The current study involved an analysis of 182 FFPE samples of ovarian tissues, ranging from the benign and borderline states to overt primary malignancies. FFPE tissue samples present an invaluable resource housed within the inventories of pathology departments and hospitals worldwide. The advantages of these samples are their conservative sizes and detailed associated clinical data, which make them an excellent choice for molecular studies. Advances in molecular techniques have refined the methodology of DNA analysis from these samples, permitting more reliable investigations into genomic aberrations related to pathologies like cancer [42]. Indeed, while RNA extraction from this type of sample has traditionally been challenging due to concerns of degradation or the modification of RNA, several studies have illustrated the potential of RNA sequencing in FFPE samples [43,44]. This creates a promising opportunity to expand the scope to not only DNA mutations but also to the transcriptional and post-transcriptional levels, thereby providing a more comprehensive genetic landscape [45].

The specified lncRNA genotypic frequency disparities were scrutinized between those with low-grade epithelial ovarian tumors, which included benign and borderline tumors, and those with malignant tumors. Furthermore, additional differences were explored within the malignant category, specifically contrasting high-grade epithelial tumors with malignant epithelial tumors, excluding high-grade serous carcinomas.

Our comparative analysis of the current patients’ pathological data revealed a remarkable distinction between the low-grade and malignant epithelial tumor groups concerning the expression profiles of specific proteins, such as HER2 and P53. These proteins are particularly noteworthy given their essential roles in cell proliferation and tumor suppression [46]. The HER2 staining was predominantly negative in the low-grade tumors. However, the 3+ staining intensity, indicative of the highest presence of HER2, was disproportionately higher in malignant tumors, confirming the association of HER2 overexpression with malignancy in epithelial OC. This receptor tyrosine kinase is known for its function in cell growth and differentiation, and the overexpression of HER2 stimulates aggressive cell proliferation, potentially increasing the tumor gradation [47]. Simultaneously, there were substantial differences in the P53 protein staining between the low-grade and malignant epithelial tumor groups. As one of the most critical tumor suppressors in human biology, alterations in P53, often through mutations, can contribute to varying tumor behavior [48].

Given the roles of these molecular entities and their contributions to cell cycle control, it is not surprising that their levels are significantly associated with pathological distinctions between the low-grade and malignant tumor groups. This also underlines the relative roles that the proteins might play in the biology and potential aggressiveness of the tumors. A significant distinction was also noted in the KRAS protein staining between the two study groups. However, the EGFR protein staining differences were not convincing enough regarding its potential influence on the pathological nature of this type of tumor. This lends further strength to the importance of understanding tumor heterogeneity. KRAS is notable, as it encodes a protein involved in transmitting chemical signals within cells and plays an essential part in cell division, differentiation, and apoptosis [49]. A significant difference in the KRAS staining suggests the potentially transformative role of KRAS in the pathophysiology of ovarian cancer conditions [50]. However, our findings did not reveal any significant distinctions concerning the staining of the EGFR protein, in agreement with Mehner et al.’s evaluation of their patient cohort and literature review, concluding that “IHC staining for EGFR might not provide a reliable prognosis indicator for patients with OC”.

The gene mutation screening of the analyzed samples revealed the presence of *BRAF* V600 and *KRAS* exon 12 mutations. No significant differences were found for these mutations between the two study groups, casting doubt on the role of these particular mutations in the divergent behavior of these ovarian tumors. In contrast, the analysis for KRAS exon 13 revealed no mutations in any of the cases, which reinforces the distinct pathophysiological trajectories in cancer [51].

LncRNAs have been found to play a crucial role in the tumorigenesis and cancer progression of various cancers, including OC [52], and they may offer new insights into potential therapeutic targets [53]. Studies have shown that lncRNAs can interact with DNA, protein, and RNA, regulating gene expression through various mechanisms [10,54,55,56]. In ovarian cancer, more than 30 lncRNAs have been identified as predictors of survival and/or the treatment response [57].

The impact of lncRNA polymorphisms on ovarian cancer is starting to emerge, with several studies providing clues as to how lncRNAs could modulate the susceptibility to this disease. For instance, a “genome-wide association study (GWAS)” conducted by Permuth et al. [58] identified several lncRNAs, including MIR2052HG, which were associated with the risk of ovarian cancer. The study involved more than 25,000 women of European ancestry, and the authors identified nine new ovarian cancer risk loci, four of which contained lncRNA genes. Among these loci, MIR2052HG stood out as the most promising candidate for ovarian cancer susceptibility. The authors showed that an lncRNA variant, rs1859962, located in the promoter region of MIR2052HG, was significantly associated with the risk of developing ovarian cancer. Furthermore, the rs1859962 variant showed a relatively high minor allele frequency (MAF) in European populations, suggesting it could be a widespread risk factor. Another study conducted by Wu et al. [59] showed that the rs4759314 and rs7958904 genetic variations in the lncRNA HOTAIR were significantly associated with epithelial OC susceptibility. The rs7958904 C allele was significantly associated with a decreased risk compared to the G allele. The investigators concluded that HOTAIR genetic variants could be a valuable biomarker for OC susceptibility and/or early disease diagnosis.

In the current study, two angio-lncRNA genetic variants were selected for investigation under different genetic models in ovarian tumors. We found, for the first time, that the MIAT rs1061540 variant was associated with the risk of malignant epithelial OC and the tumor grade. More specifically, the MIAT TT genotype conferred a lower risk for ovarian tumorigenesis under the homozygous comparison, and the “T” allele carriers were less likely to have high-grade serous carcinoma under the heterozygous/homozygous comparison models, which was also confirmed via Cox regression analysis. However, MALAT rs3200401 did not correlate significantly with ovarian tumorigenesis or the grade in the present cohort.

While the cellular implications of MIAT in multiple disorders remain to be identified, several studies have proposed this type of lncRNA as a promising molecular marker, as concluded by Aznaourova et al. [60]. MIAT has been found to regulate the expressions of various genes implicated in cell proliferation, angiogenesis, apoptosis, and differentiation [61]. Although the currently studied variant rs1061540 has not been previously associated with cancer, other MIAT-related SNPs have been associated with cancer risk [62]. For instance, Zheng et al. found that the C allele of rs1061451T/C is a protective factor against non-small-cell lung cancer, and MIAT could act as competitive endogenous RNA (ceRNA), which compete with protein-coding mRNAs for binding to miRNAs [57] via miR-133a-5p, modulating the *MYO1B*, *WNT9A*, and *SGK1* gene expression levels [63]. Further studies are needed to elucidate the precise role of MIAT rs1061540 in cancer and its potential as a biomarker for cancer risk assessment.

The association of the MALAT1 rs3200401 variant with cancer risk has been extensively investigated in recent years. For instance, it was found that females with an rs3200401C/T genotype had a lower risk of breast cancer [64], and T allele carriers had a better survival for advanced lung adenocarcinoma [65] and a lower risk of prostate adenocarcinoma in a Ukrainian Population [66] than “C” allele carriers. Also, individuals with the TT genotype were associated with an increased risk of oral squamous cell carcinoma [67], esophageal squamous cell carcinoma in the Chinese population [68], and gastric cancer in male Korean patients under heterozygous and dominant models [69]. However, consistent with our findings, there was no significant association between rs3200401C/T and hepatocellular carcinoma [70,71] or cervical cancer in Taiwanese women [72]. Additionally, a recent meta-analysis conducted by Li et al. found no significant association between the MALAT1 rs3200401C/T variant and the overall cancer risk. However, it may be linked to a higher risk of colorectal cancer, which needs more studies for further validation [73]. Other MALAT1 functional variants have shown associations with the risk of cancer, for example, colorectal cancer (rs619586, rs664589, and rs1194338) and hepatocellular cancer (rs619586), as confirmed by the recent meta-analysis conducted by Cao et al. [74]. It is worth noting that the discrepancies between different studies and ours could be due to the ethnic differences, variable sample sizes, types of study populations (hospital-based vs. general population), study designs, and types of cancer, among others.

Although this study is the first, to the best of our knowledge, to identify the association of angio-lncRNAs with ovarian tumorigenesis and the grade, some limitations should be considered. For example, the number of included OC samples is relatively small, and the applicability of the findings to other populations might be limited. The retrospective study design could limit the ability to determine cause and effect. The selected variants are representative polymorphisms that do not cover all the potential candidate SNPs. Also, RNA analysis could potentially provide rich data, giving way to novel insights into our investigation and exploring, in part, the putative mechanism through which the studied variants could confer the OC risk. Additionally, as wide-ranging studies suggest, genome-wide analyses and their integration with the current approach could further augment and inform our understanding of the genetic landscape in OC. These extensive and comprehensive tools can provide insights into genomic variations, epigenetic modifications, and gene–environment interactions, thereby enabling a more holistic view of tumor behavior beyond the scope of individual genes or loci [75,76,77,78]. Also, the other potential confounding factors that might affect the association between the studied lncRNA variants and OC risk or progression should be considered. In this sense, further prospective research is warranted to validate the consistency of our results in a large sample and across different ethnic populations. Also, gene expression and functional studies should be conducted to shed light on the association of the study variants and other potential lncRNA-related polymorphisms with the OC risk.

## 4. Materials and Methods

### 4.1. Archived Tissue Sampling

This retrospective study included 182 specimens of females with benign, borderline, and primary malignant ovarian tissues collected from governmental hospitals in Khartoum State, Sudan. The archived “formalin-fixed paraffin-embedded (FFPE)” samples of resected tissue specimens and the retrieved clinicopathological data during the last eight years were included in the present work. None of the patients had a history of receiving any neoadjuvant chemotherapy or radiotherapy before sampling. Complete clinicopathological data (including patients’ age, tumor size, staging, etc.) were obtained from patient medical records. Samples that were not homogeneous and/or histologically well characterized (determined by an experienced pathologist, A.T.I.) were excluded. The study was conducted following the guidelines in the Declaration of Helsinki and was approved by the “Ethical Committee at the Ministry of Health, Khartoum State, Sudan; 1/4/2015”. Informed consent of the patients was not applicable as the study was a retrospective one, including archived FFPE samples.

### 4.2. Pathological Assessment

Ovarian epithelial tumors were classified histologically according to the World Health Organization (WHO) classification of Female Genital Tumors [79] into serous, mucinous, endometrioid, and Brenner tumors. Each histological type is subdivided into benign, borderline, and malignant. For malignant serous tumors, high-grade serous carcinoma was defined as serous tumors with high-grade atypia and mitosis ≥ 12/10 hpf with mutant p53 (all or null pattern). Ovarian mucinous carcinoma was diagnosed as primary ovarian mucinous carcinoma after excluding metastatic mucinous tumors clinically, radiologically, and histologically. It is worth noting that, due to the incomplete availability of data collected from surgical exploration during the time of operation and CT scan results for most of the cases, the authors could not apply the FIGO staging system related to OC cases [80]. Data on all immunohistochemistry (IHC) staining for the p53, “epidermal growth factor receptor (EGFR)”, “human epidermal growth factor receptor 2 (HER2)”, and “Kirsten rat sarcoma viral oncogene homolog (KRAS)” were retrieved from patient files. Also, the available molecular data related to the BRAF and KRAS 12 and 13 codon mutations for the included specimens were recruited via personal communication with lab colleagues.

### 4.3. Criteria for Selecting the lncRNA SNPs and In Silico Data Analysis

A literature review and bioinformatic analyses were applied to select the common (i.e., MAF ≥ 0.1) SNPs in the specified lncRNA genes. The genomic structures and variants of the MALAT1 and MIAT genes were retrieved from the “Ensembl Genomic database (www.ensembl.org)”. After list sorting, the most common biallelic variants, MALAT rs3200401 (C/T) and MIAT rs1061540 (C/T), were selected. The putative regulatory roles of these non-coding variants mediated by their 3D genome topology and their 3D interactions with other genes/variants mediated via chromatin loops were retrieved from the non-coding genomic variant annotation database (https://omic.tech/3dsnpv2/) [81] and visualized as 3D interacting genes, regulatory enhancers, promotors, transcriptional factors, and conservation scores. Also, tissue-specific predictions of the regulatory probabilities of the SNPs of interest on the provided genes were calculated using “RegVar” [82]. Prior publications were retrieved from the “human gene database GeneCards (www.genecards.org)” and the “National Center of Biotechnology Information; NCBI (https://www.ncbi.nlm.nih.gov/)” [83] (all databases last accessed on 20 March 2023).

### 4.4. Analysis of LncRNA Gene Variants MALAT1 rs3200401 and MIAT rs1061540 

Genomic deoxynucleic acid (gDNA) was purified from the FFPE tissue specimen (5 sections each with 8 µm thickness) using the QIAamp DNA FFPE tissue kit (Qiagen, Hilden, Germany), according to the manufacturer’s procedure. The isolated nucleic acid purity and concentrations were evaluated using the “NanoDrop ND-1000”(NanoDrop Tech., Inc., Wilmington, DE, USA). Furthermore, the DNA integrity was checked by running 100 ng/sample on a 1.0% agarose gel electrophoresis. Genotyping for the selected variants was assayed using real-time PCR allelic discrimination technology, as described in our previous works [34,35,84,85,86]. Briefly, PCR reactions were run blindly to the types of samples in a final volume of 25 µL, containing gDNA (20 ng), TaqMan Universal PCR Master Mix, and TaqMan SNP Genotyping Assay Mix, according to the standard protocols. The specific assay for the MALAT1 variant (C___3246069_10; Cat. #4351379) detects the transition substitution T/C in the target context sequence (VIC/FAM): GAATGCAGTTGTCTTGACTTCAGGT[T/C]TGTCTGTT CTGTTGGCAAGTAAAT. Meanwhile, that of the MIAT variant (C___2467719_1_; Cat# 4351379) detects the transition substitution C/T in the target context sequence (VIC/FAM): GACTTTAGATGCATTTTTCTCAGTG[C/T]AAGTGTCTGAGCTCATCTC CAGTTC, (https://www.thermofisher.com/; last accessed 30 March 2023). No-template-control (NTC) samples that do not contain DNA templates were used as negative controls in each run to show the background signal and detect the contaminations (if any) that might give false-positive signals. The “StepOne™ Real-Time PCR System” (Applied Biosystems, USA) was used for PCR amplification as follows: (1) an initial hold at 95 °C (10 min), followed by (2) a 40-cycle two-step PCR denaturation at 95 °C (15 s) and annealing/extension at 60 °C (1 min) [29,30,31]. SDS software version 1.3.1 (Applied Biosystems) was used for allelic discrimination calls. About 10% of randomly selected samples were re-genotyped in separate runs to exclude the potential false genotype calls, with 100% yielding concordance results.

### 4.5. Statistical Analysis

The SPSS for Windows (version 27.0) and R (version 3.5.3) tools were applied for data analysis. G*Power (version 3.1.9.2.) was used to calculate the study power. By selecting an effect size of 0.37, at a 95% significance level, and with a minimal sample size required to reject the null hypothesis (N = 182), this study’s power was 91%. Genotype/allele frequencies and genetic association models [87] were analyzed using SNPStats (https://www.snpstats.net/start.htm) (accessed 20 February 2023). Multivariable regression models were adjusted by patient’s age at diagnosis and presented as odds ratios (ORs) and 95% confidence intervals (CIs). The impact of the studied MALAT1 and MIAT variants on the OC risk was tested using two different measurements: the “Akaike Information Criterion (AIC)” and “Bayesian Information Criterion (BIC)”. These two statistical measures compare and select the best-fitting model among a set of candidate models for a given dataset. Both the AIC and BIC consider the goodness of fit and the complexity of the model. The AIC, developed by Hirotugu Akaike, is defined as AIC = −2 × log(likelihood) + 2 × K, where “likelihood” represents the likelihood of the model given the data, and “K” denotes the number of parameters in the model. The model with the lowest AIC value is considered the best fit for the data. The BIC, proposed by Gideon E. Schwarz, is defined as BIC = −2 × log(likelihood) + K × log(n), where “likelihood” and “K” are the same as in the AIC, and “n” represents the sample size. Like the AIC, the model with the lowest BIC value is considered the best fit for the data. The main difference between the AIC and BIC lies in the penalty term for model complexity. The BIC incorporates a more substantial penalty for model complexity, as it considers the sample size, whereas the AIC only considers the number of parameters. This penalty difference can lead to different model selections, with the BIC generally favoring simpler models compared to the AIC. A two-sided chi-square test was applied for qualitative variables. Significance was set at *p* < 0.05.

## 5. Conclusions

While the full implication of the lncRNAs MIAT rs1061540 and MALAT1 rs3200401 in ovarian cancer development remains to be determined, it is clear that MIAT rs1061540 played a role in modulating the risk of this disease in the present study cohort. While our present study is a step in the direction of elucidating the genetic dynamics in ovarian cancer, a broader approach could potentially unravel more complex and interconnected facets of the disease. Further research in this area is warranted to improve our understanding of ovarian cancer biology and develop novel lncRNA-based diagnostic strategies.

## Figures and Tables

**Table 1:** Demographic and pathological characteristics of the study population.

Characteristics	Total Tumors (*N* = 182)	Low-Grade Epithelial Tumors (*N* = 85)	Malignant Epithelial Tumors (*N* = 97)	*p*-Value
Demographic data				
Age in years				
<30 years	34 (18.7)	26 (30.6)	8 (8.2)	**0.001**
30–49 years	96 (52.7)	38 (44.7)	58 (59.8)	
≥50 years	52 (28.6)	31 (32.0)	21 (24.7)	
Pathological subtype				
Benign epithelial tumors	72 (39.6)	72 (84.7)	0 (0.0)	**<0.001**
Borderline epithelial tumors	13 (7.1)	13 (15.3)	0 (0.0)	
High-grade serous carcinoma	49 (26.9)	0 (0.0)	49 (50.5)	
Other than high-grade serous	48 (26.4)	0 (0.0)	48 (49.5)	
Immunohistochemistry staining				
HER2 protein staining				
Negative	97 (53.3)	74 (87.1)	23 (23.7)	**<0.001**
1+	24 (13.2)	6 (7.1)	18 (18.6)	
2+	27 (14.8)	4 (4.7)	23 (23.7)	
3+	34 (18.7)	1 (1.2)	33 (34)	
P53 protein staining				
Negative	134 (73.6)	85 (100)	49 (50.5)	**<0.001**
Positive *	48 (26.4)	0 (0.0)	48 (49.5)	
KRAS protein staining				
Negative	97 (53.3)	35 (41.2)	62 (63.9)	**0.003**
Positive	85 (46.7)	50 (58.8)	35 (36.1)	
EGFR protein staining				
Negative	175 (96.2)	83 (97.6)	92 (94.8)	0.45
Positive	7 (3.8)	2 (2.4)	5 (5.2)	
Gene mutation screening				
*BRAF* V600				
Wild	16 (8.8)	9 (10.6)	7 (7.2)	0.24
Heterozygote	154 (84.6)	73 (85.9)	81 (83.5)	
Mutant	12 (6.6)	3 (3.5)	9 (9.3)	
*KRAS* exon 12				
Wild	28 (15.4)	15 (17.6)	13 (13.4)	0.54
Mutant	154 (84.6)	70 (82.4)	84 (86.6)	
*KRAS* exon 13				
Wild	182 (100)	85 (100)	97 (100)	NA

Values are shown as numbers (%). The chi-square/Fisher exact tests were used. * Positive: mutant p53 immunostaining is defined as “the combination of more than one pattern of staining (e.g., wild-type and one or more mutant patterns or two different mutant patterns), with each present in at least 5% of tumor cells”, according to Park et al. [40]. The bold values indicate significance at a *p*-value < 0.05. HER2: human epidermal growth factor receptor 2; KRAS: Kirsten rat sarcoma viral oncogene homolog; EGFR: epidermal growth factor receptor; NA: Not applicable.

**Table 2:** Association of demographic data, molecular mutations, and protein markers according to the grade of ovarian tumors.

Characteristics	Levels	High-Grade Serous Carcinoma (*N* = 49)	Other than High-Grade Serous (*N* = 48)	*p*-Value
Demographic data				
Age in years	<30 years	2 (4.1)	6 (12.5)	0.27
	30–49 years	32 (65.3)	2 (54.2)	
	≥50 years	15 (30.6)	16 (33.3)	
Immunohistochemistry staining				
HER2 protein staining	Negative	11 (22.4)	12 (25)	0.28
	1+	8 (16.3)	10 (20.8)	
	2+	9 (18.4)	14 (29.2)	
	3+	21 (42.9)	12 (25)	
P53 protein staining	Negative	0 (0.0)	48 (100)	**<0.001**
	Positive *	49 (100)	0 (0.0)	
KRAS protein staining	Negative	30 (61.2)	32 (66.7)	0.67
	Positive	19 (38.8)	16 (33.3)	
EGFR protein staining	Negative	45 (91.8)	47 (97.9)	0.36
	Positive	4 (8.2)	1 (2.1)	
Gene mutation				
*BRAF* V600	Wild	1 (2.1)	6 (12.2)	0.15
	Heterozygote	42 (87.5)	39 (79.6)	
	Mutant	5 (10.4)	4 (8.2)	
*KRAS* exon 12	Wild	11 (22.9)	2 (4.1)	**0.007**
	Mutant	37 (77.1)	47 (95.9)	
*KRAS* exon 13	Wild	49 (100)	48 (100)	NA

Values are shown as numbers (%). The chi-square/Fisher exact tests were used. * Positive: mutant p53 immunostaining is defined as “the combination of more than one pattern of staining (e.g., wild-type and one or more mutant patterns or two different mutant patterns), with each present in at least 5% of tumor cells”, according to Park et al. [40]. The bold values indicate significance at a *p*-value < 0.05. HER2: human epidermal growth factor receptor 2; KRAS: Kirsten rat sarcoma viral oncogene homolog; EGFR: epidermal growth factor receptor; BRAF: v-RAF murine sarcoma viral oncogene homolog B1; NA: Not applicable.

**Table 3:** Genetic association models for the impact of MALAT1 on ovarian cancer risk.

Model	Genotype	Low-Grade Epithelial Tumors (N = 85)	Malignant Epithelial Tumors (N = 97)	OR (95% CI)	*p*-Value	AIC	BIC
Codominant	C/C	31 (36.5%)	35 (36.1%)	1	0.43	251	263.9
	C/T	43 (50.6%)	44 (45.4%)	0.93 (0.48–1.79)			
	T/T	11 (12.9%)	18 (18.6%)	1.64 (0.66–4.09)			
Dominant	C/C	31 (36.5%)	35 (36.1%)	1	0.83	250.7	260.3
	C/T-T/T	54 (63.5%)	62 (63.9%)	1.07 (0.58–1.98)			
Recessive	C/C-C/T	74 (87.1%)	79 (81.4%)	1	0.20	249.1	258.7
	T/T	11 (12.9%)	18 (18.6%)	1.71 (0.74–3.93)			
Overdominant	C/C-T/T	42 (49.4%)	53 (54.6%)	1	0.46	250.2	259.8
	C/T	43 (50.6%)	44 (45.4%)	0.80 (0.44–1.45)			

Binary logistic regression was performed and adjusted by patient age. OR (95% CI): odds ratio (95% confidence interval); AIC: Akaike Information Criterion; BIC: Bayesian Information Criterion.

**Table 4:** Genetic association models for the impact of MIAT on ovarian cancer risk.

Model	Genotype	Low-Grade Epithelial Tumors	Malignant Epithelial Tumors	OR (95% CI)	*p*-Value	AIC	BIC
Codominant	C/C	32 (37.6%)	55 (56.7%)	1	**0.040**	246.3	259.1
	C/T	31 (36.5%)	27 (27.8%)	0.53 (0.27–1.05)			
	T/T	22 (25.9%)	15 (15.5%)	**0.39 (0.18–0.88)**			
Dominant	C/C	32 (37.6%)	55 (56.7%)	1	**0.014**	244.7	**254.3**
	C/T-T/T	53 (62.4%)	42 (43.3%)	**0.47 (0.26–0.87)**			
Recessive	C/C-C/T	63 (74.1%)	82 (84.5%)	1	0.08	247.6	257.2
	T/T	22 (25.9%)	15 (15.5%)	0.51 (0.24–1.08)			
Overdominant	C/C-T/T	54 (63.5%)	70 (72.2%)	1	0.28	249.5	259.2
	C/T	31 (36.5%)	27 (27.8%)	0.70 (0.37–1.33)			

Binary logistic regression was performed and adjusted by patient age. OR (95% CI): odds ratio (95% confidence interval); AIC: Akaike Information Criterion; BIC: Bayesian Information Criterion. Bold values indicate significance at *p* < 0.05.

**Table 5:** Frequency of genotype combination and risk of ovarian cancer.

	MALAT1	MIAT	Total	Low-Grade Epithelial Tumors	Malignant Epithelial Tumors	OR (95% CI)	*p*-Value
1	C	C	0.407	0.4	0.423	1	---
2	T	C	0.23	0.159	0.283	1.67 (0.90–3.11)	0.11
3	C	T	0.195	0.217	0.164	0.78 (0.45–1.37)	0.39
4	T	T	0.168	0.224	0.13	0.65 (0.36–1.20)	0.18

OR (95% CI): odds ratio (95% confidence interval). Significance was set at *p* < 0.05.

## Data Availability

All datasets presented in this study are included in the article/supplementary material.

## Supplementary Materials

The following supporting information can be downloaded at https://www.mdpi.com/article/10.3390/epigenomes8010005/s1, Table S1: Tissue-specific predictions of regulatory probabilities of the study variants. Figure S1: Color scheme of 15 chromatin states in ChromHMM core model applied in Circos plot. Figure S2: Color scheme of twelve cell types for the chromatin loop track in Circos plot.
