# Supplementary material for: Angio-Long Noncoding RNA MALAT1 (rs3200401) and MIAT (rs1061540) Gene Variants in Ovarian Cancer
Source: Epigenomes. 2024 Jan 29;8(1):5. doi: 10.3390/epigenomes8010005 (PMC10885055; doi:10.3390/epigenomes8010005)
Supplement: Supplementary file 1 [file epigenomes-08-00005-s001.zip › epigenomes-2752395-supplementary.pdf]

Angio-long noncoding RNAs-related gene variants in ovarian cancer

COLOR	STATE NO.	MNEMONIC	DESCRIPTION
	1	TssA	Active TSS
	2	TssAFlnk	Flanking Active TSS
	3	TxFlnk	Transcr. at gene 5′ and 3′
	4	Tx	Strong transcription
	5	TxWk	Weak transcription
	6	EnhG	Genic enhancers
	7	Enh	Enhancers
	8	ZNF/Rpts	ZNF genes & repeats
	9	Het	Heterochromatin
	10	TssBiv	Bivalent/Poised TSS
	11	BivFlnk	Flanking Bivalent TSS/Enh
	12	EnhBiv	Bivalent Enhancer
	13	ReprPC	Repressed PolyComb
	14	ReprPCWk	Weak Repressed PolyComb
	15	Quies	Quiescent/Low

Figure S1. Color scheme of 15 chromatin states in ChromHMM core model applied in CIRCOS plot. Data source (https://omic.tech/3dsnpv2/) (last accessed 30 March 2023) [32].

COLOR	CELL TYPE	DESCRIPTION	TISSUE
	GM12878	Lymphoblastoid Cells	Blood
	K562	K562 leukemia Cells	Blood
	H1-hESC	Embryonic stem cells	ESC
	IMR90	Fetal lung fibroblasts	Lung
	HeLa-S3	Cervical carcinoma cells	Cervix
	HUVEC	Umbilical vein endothelial cells	Blood vessel
	NHEK	Epidermal keratinocytes	Skin
	HMEC	Mammary epithelial cells	Breast
	KBM-7	Chronic myelogenous leukemia (CML) cells	Blood
	LNCaP	Prostate adenocarcinoma	Prostate
	PC3	Prostate cancer cells	Prostate
	PrEC	Prostate epithelial cell line	Prostate

Figure S2. Color scheme of twelve cell types for the chromatin loop track in CIRCOS plot. Data source (https://omic.tech/3dsnpv2/) (last accessed 30 March 2023) [32].

Table S1. Tissue-specific predictions of regulatory probabilities of the study variants

SNP ID	SNP Position	Gene Symbol	Gene Position	Distance	Tissue	Score
rs3200401	chr11:65271831	MALAT1	chr11:65265232	6599	Ovary	0.21942
rs1061540	chr22:27062037	MIAT	chr22:27042391	19646	Ovary	0.64169

Data source https://regvar.omic.tech/ (last accessed 30 March 2023) [33].
